# High-throughput targeted SNP discovery using Next Generation Sequencing (NGS) in few selected candidate genes in *Eucalyptus camaldulensis*

**DOI:** 10.1186/1753-6561-5-S7-O17

**Published:** 2011-09-13

**Authors:** Prasad Suresh Hendre, Rathinam Kamalakannan, Rathinavelu Rajkumar, Mohan Varghese

**Affiliations:** 1 ITC R&D Centre, Peenya Insdustrial Area, No.3, 1st Main, 1st Phase, Bangalore- 560 058, Karnataka, India

## Background

The present era of high throughput technologies offer immense promise and innovative applications for SNP discovery and high quality parallel genotyping [1,2]. Using advancements in the next generation sequencing (NGS) technologies, the *en masse *SNP discovery for targeted genomic regions is possible for eucalypts. The river red gum or *Eucalyptus camaldulensis* (*Ec*) is a fast growing, hardy and highly adaptable eucalypt species acclimatized to Indian climatic conditions and these new advancements would aid in developing new tools and techniques for its improvement. In our knowledge, limited efforts have been undertaken to identify SNP markers in eucalypts either by employing RNA sequencing [3] or by using few genes available in the literature [4]. Despite these miniscule efforts, useful SNP markers were discovered in *Cinnamoyl CoA Reductase* (*CCR*) gene with potential application [5]. Using the recently released whole genome sequence of *E. grandis* (*Eg*), herein we describe targeted SNP discovery in 41 candidate genes by employing Illumina’s 72-bases paired end sequencing technology.

## Materials and methods

**Figure 1:**
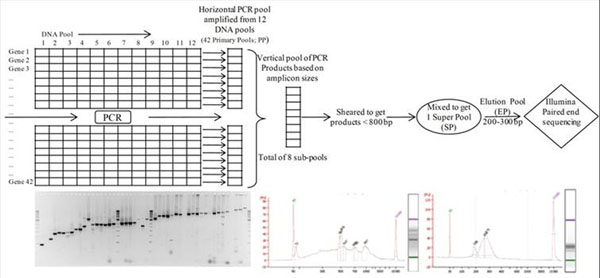
Stategy for hierarchical pooling of 96 DNAs and PCR products for SNP discovery using Illumina NGS platform

The DNA was isolated from a SNP discovery panel consisting 96 individuals from a naturally mating *Ec* population from Australia following standard procedures (modified CTAB method). Twelve primary DNA pools were constituted by mixing equimolar concentrations of eight DNAs @ 10 ng/mL. Forty one genes selected for SNP discovery were identified from *Eg* genome (http://eucalyptusdb.bi.up.ac.za/gbrowse8x) by employing Arabidopsis TAIR 9 gene IDs. Further the primer pairs were designed to amplify the gene fragments. The individual primary DNA pool was amplified (Veriti-ABI) using *Paq* DNA polymerase (Agilent Technologies), all amplicons pooled (figure 1), eluted if necessary (*Ec*CRE-AHK4, *Ec*OBP1), precipitated using ethanol and dissolved in TE (0.1).

A paired end library suitable for 72-bases read length was prepared and sequenced on an Illumina GAIIx sequencer and analyzed using bwa and samtools with appropriate parameters (outsourced to Genotypic Technologies Ltd, Bangalore). The SNP data was adjusted for read depth (1/10^th^ SD) and rare allele frequency (<5%). Further approximate equal frequency (EF) blocks were manually estimated by nearest neighborhood (NN) analysis in MS Excel (MS Office 2007), wherein, a block of NN SNPs having frequency difference of less than 0.02-0.03 was considered as single EF block. Web-based gene prediction tool FGENESH (http://linux1.softberry.com) was used for identifying genic regions such as UTRs, exons and introns with *Arabidopsis thaliana* gene model.

## Results and discussion

**Figure 2:**
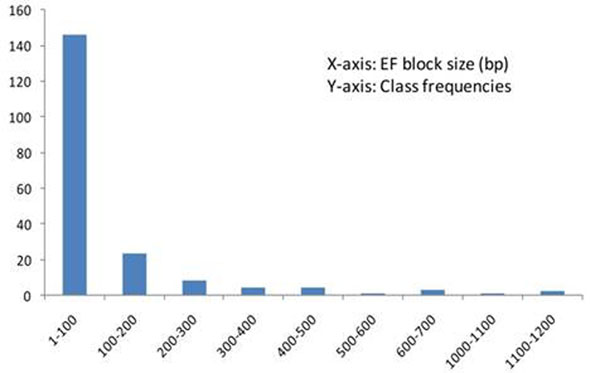
Bar graph showing distribution of 198 frequency (EF) blocks according to their length (bp)

Forty one growth and adaptive genes were selected based on literature search [6, TAIR database]. A total of 100.5 kb genomic sequence from *Ec* genome spread over ~1055 Mbp reads was generated (~94% high quality reads with average read depth 6124). A total of 11,329 SNPs were polymorphic within *Ec* and 378 SNPs exhibited inter-species polymorphism between *Ec* and *Eg*. In addition, 75 insertions and 90 deletions within *Ec* and eight intra-specific deletions in comparison to *Eg* were detected. After appropriate corrections as described, the ‘useful’ SNP number reduced to 1,191 which was ~10.5% of the original SNP count (~frequency of 1 per 84.5 bp). Table 1 describes findings from the present analysis of SNPs. A total of 198 putative EF blocks containing 541 SNPs, grossly comparable to LD blocks, with 55, 65 and 34 in exons, introns, exon-intron junctions respectively were detected (rest all were small in numbers) with an average length of ~105 bp (SD: ± 182; range: 1-1234 bp, distribution shown in figure 2; ~3 SNPs/block) and would aid in selection of SNPs. The comparable mean lengths adjusted for the respective amplicon lengths were around 0.014 to 0.016 (SD: ±0.013 to ±0.015) for exons, introns and nongenic regions whereas for intron-exon junctions it was 0.028±0.023, significantly longer than the rest (p=0.03).

**Table 1:** Results from SNP discovery in 41 candidate genes.

***Predicted gene region***	SNP frequency parameters			SNP classification			
	
	***SNP count (range)***	***SNP Frequency in bases/SNP (range)***	***Total length in bp (range)***	***Ts***	***Tv***	***Total***	***Ratio(Ts/Tv)***
***5'UTR(n=1)***	4	40.8	163	1	3	4	0.33

***Exons(n=176)***	427 (1-52)	105.8 (0-1339)	45,177 (200-3487)	310	117	427	2.65

***Introns(n=136)***	536 (0-64)	71.3 (0-472)	38,210 (68-5079)	332	204	536	1.63

***3'UTR(n=27)***	54 (0-11)	81.6 (0-358)	4,405 (7-674)	33	21	54	1.57

***Unclassified(n=13)***	69 (0-25)	62.7 (0-425)	4,329 (4-425)	40	29	69	1.38

***Nongenic(n=17)***	101 (0-25)	82.6 (0-974)	8,340 (6-1481)	63	38	101	1.66

***Total(n=370)***	1,191 (1-115)	84.5 (38.2-974)	100,624 (634-9864)	779	412	1191	1.89

**Note**: n: number of units detected from 41 genes; Ts: transitions, Tv: transversions

## Conclusions

Herein, NGS (Illumina) platform was successfully used for identifying ~1,200 SNPs in 41 targeted genes in *Ec* which has shed important light on quantitative and qualitative distribution of SNPs. In addition, the analysis of EF blocks also provided important guidelines for selection of SNPs for genotyping.
